
JOURNAL INFORMATION
==============================
NLM Title Abbreviation: Med Chir Rev
NLM Journal ID: 0034617

The Medico-Chirurgical Review

Publisher: Biomedical Journal Digitization Project

ARTICLE INFORMATION
==============================
PMCID: PMC4966267
PMID: 29917637
Article version: 1
Subjects: Reviews, Diseases Produced by Malaria

On Difficult Cases of Parturition; and the Use of Ergot of Rye

Print publication date: 1828 Oct 1
Volume: 9
Issue: 18
First page: 358
Last page: 360
1828 Aug
License: This work is free of known copyright restrictions. For more information, please see PMC Back Issue Digitization.
License URL: https://creativecommons.org/publicdomain/mark/1.0/

==============================

On perforation from chronic ulceration of the stomach we need not dwell. The symptoms of sudden peritonitis generally supervene, and death soon closes the scene. We have now finished the first fasciculus, and laid before our readers a very ample analysis and numerous extracts, by which they can judge for themselves. Did we indulge in rigid censure or indiscriminate praise we might be suspected, and justly so, of belonging to one of the parties described in the first page of this paper.

We shall proceed to other organic diseases of the stomach in our next article.

---

### IX.

*On difficult Cases of Parturition: and the Use of Ergot of Rye.* By W. MICHELL, Member of the Royal College of Surgeons. Octavo, pp. 128. Underwoods, 1828.

AMONG the blessings and curses attendant upon, or rather produced by, the progress of civilization, are crooked spines and lingering labours. Well! had it not been for the evil sent into this world, we should have been deprived of much of the good we possess. The diseases of the body have called forth the energies of the mind, and for every corporeal defect we have twenty different remedies. It is curious that the action of *specific* medicines (by which we mean medicinal substances that act *specifically*, or particularly on one organ or function) should have been more frequently discovered by the ignorant, or at least the empirical, than by the most enlightened and learned physicians. The reason, however, is not very difficult to assign. The ignorant look upon all medicinal agents as specifics, and consequently are always hunting after such agents. The LEARNED have little faith in specifics, and are more employed in the investigation of structures, functions, and the general operations of drugs on the animal economy. We believe there is no substance better entitled to the title "SPECIFIC" than the ergot of rye—a substance, the good properties of which were first appreciated by old women beyond the Atlantic. If we can credit the anticipations of the author whose work is before us, and who appears to be a sensible, a well-informed, and an experienced practitioner, this little abortive grain of rye will effect a greater revolution in medical practice than vaccination—and will go farther to bring back midwifery within the domain of its original professors, than all the orations of Sir Anthony Carlisle. The following extract will make our meaning intelligible.

"Every candid and honest practitioner will admit, that if we could discover any treatment which would effect delivery with safety to the mother and child, it would be preferable in other respects, and tend to the comfort of the Medical Practitioner, to resign the Midwifery branch again to the Female Attendant, except in some particular dangerous cases. To increase and quicken the Uterine action, and thereby facilitate delivery, I have tried various vegetable and mineral substances. Many of the vegetables have possessed this power to a certain extent, but the minerals produced no effect whatever. These endeavours led me immediately to notice the accounts of the Ergot of Rye, and to subject its pretensions to the test of my own experience. The result has been, that after its successful application for many years, I am now fully convinced that it is a safe and efficacious medicine in accouchery cases; possessing all the properties which a Practitioner could desire. Its effect on the contraction of the Uterus, both before the expulsion of the Fœtus, and after this has been accomplished, is such as to remove all danger attendant on the woman, quickening and facilitating the delivery, and preventing spasm or flooding afterwards. I am of opinion, therefore, that as soon as it is generally known in female practice, it will supersede the necessity for male practitioners, except in a very few instances, where the disproportion of parts is very great, or the presentation such that delivery cannot be effected without turning; in such cases the medical man will still be called in, but these will not occur in more than one out of 200 of the medical man's present accouchery patients. This, I have no doubt, will be hailed as a great relief by the generality of the Surgeon Apothecaries in country practice. It may be considered as somewhat extraordinary, that the virtues of the Ergot should not have been known until the present century; had they been discovered before, the Surgeon would never have acquired his present practice in Midwifery—the Females would still have retained it, and the lever and forceps would never have been invented—the new practice would not have superseded the old, which was a far preferable system, and most unquestionably so now, when we have the Ergot to assist in cases of sluggish labour, from rigidity of the Ligamentous, and other soft parts of the Pelvis. Before the Ergot was administered, the forceps were a necessary appendage of Midwifery practice; indeed, without them the surgeon could scarcely have superintended this practice together with his other engagements. When the Os Uteri was fully dilated, and the friends and patient most anxious to have the delivery effected by any means in the power of the Accoucheur, it cannot be expected that with the means in his possession of expediting the labour, he should prefer waiting twenty-four hours, during which his attendance is required in various other places, rather than have recourse to the forceps which have perhaps frequently relieved him in similar circumstances. Had not the invention of the forceps by Dr. Chamberlain preceded the discovery of the virtues of the Ergot of Rye, it would have been received as the greatest boon ever given to the medical world. That its benefits have not been more generally acknowledged, may, perhaps, in a great measure, be attributed to the prejudices of self-interest, which must clearly discern a falling off of fees when Ergot shall have been extensively introduced. When rye comes into general use, the medical man will seldom be called to apply the Forceps or the Vectis, and we may then perhaps find many a Forceps in the state of those presented to the Medico Chirurgical Society by Mr. Cansardine of Woodham, Mortimer Hall, in want of fresh polishing from long neglect." 56.

Of the natural history and description of the ergot of rye, we do not deem it necessary to take much notice. Mr. M. proves that the ergot may be taken in considerable doses without detriment—he himself having swallowed eighteen drachms in fourteen days, without the least inconvenience. At the same time he thinks that half a drachm is quite sufficient for a dose. In respect to the modus operandi of the medicine, the following are our author's opinions.

"I consider it to act by contracting the muscular fibre of the extremities, and by causing a greater quantity of blood to be determined to the uterus. Its power is also said to admit of no control, to this I answer distinctly, that opium will be found to stop its effects within a very few minutes. I allow, with Dr. H., that when administered it has created astonishment, but I do not admit that its powers are but imperfectly known, as I have been in the habit of giving it for many years. It has been viewed as inert on mother and fœtus, and the only effect it has on the mother, is to cause deadness on all parts of the body, and on the child nothing more than what arises from the great pressure of the uterus. It is stated to be equivocal in its character, whereas there is no medicine that has a more decided effect, not even a purgative—it is also said not to take effect always on the uterus, but to bring on distressing symptoms—my experience has shewn me none of these, except vomiting, which is almost a certain attendant on the rapid dilatation of the os uteri. Another objection is, that when the child is born any length of time after its administration, it is always still-born. This opinion of its effects I consider to be altogether erroneous. The reason why the death of the infant has been attributed to it is, that it is only administered in long, lingering cases, in which it is well known the child is frequently still-born without the exhibition of ergot. It is asserted as a certainty, that it has destroyed life—this I do not credit: I have given it largely and frequently with the best effects, and have in no case apprehended such a result *from its use.*" 74.

The ergot has been said to put mother and fœtus in jeopardy—Mr. M. avers that it has, in his practice, "saved the lives of many children, and were it introduced into general practice, the death of a woman in child-bed would be rarely or never heard of."

"Flooding is also said to cause death when ergot is administered; from my experience, I should say, death from flooding, when ergot has been given, can never occur, it is, on the contrary, of the greatest advantage in cases of flooding, and were it only for its virtues in hæmorrhage, it would be a most invaluable medicine." 78.

We have had the means of knowing that the ergot has proved a very powerful remedy in cases of uterine hæmorrhage lately, in this metropolis, and therefore, we earnestly solicit the attention of our obstetrical brethren to this important subject. Upwards of 30 cases are detailed by the author, and he informs us, that "an abstract of those he has notes of would fill a large volume." The general dose is ℈i. in infusion, repeating this quantity according to circumstances. Mr. M. adds one third milk to the infusion, which makes it sit lighter on the stomach.

"I consider the ergot as such an acquisition to my midwifery practice, that I never go without it. I have never once regretted its use, and I invariably find that women recover much better after it than they had done before. If the woman has been subject to flooding, on former labours, I have given the ergot with a view to prevent it, with very good success. It also mitigates the severity of the after-pains, so as nearly to subdue them altogether. Its virtues are not confined to the acceleration of the action of the uterus during labour, it also causes it to contract immediately, and detaches the placenta from the body of the uterus, so that you have never to wait five minutes for its separation. I have never known an instance of adhesion when ergot has been given. I would, however, caution those who are strangers to its properties, to take away the placenta directly, otherwise they will find the uterus contracting all around it, and will experience some little trouble in the dilatation of the os uteri, I have several times found the os uteri contracting so firmly, as to oblige me to introduce the finger through it." 123.

The little volume, besides containing a very interesting dissertation on the ergot of rye, embraces the subject of difficult parturition generally, and ought to be in the library of every obstetric practitioner.
